# Interaction effect between blood selenium levels and stroke history on all-cause mortality: a retrospective cohort study of NHANES

**DOI:** 10.3389/fneur.2024.1404570

**Published:** 2024-07-05

**Authors:** Yanli Li, Lanqun Liu, Zufu Yang, Jimin Xu

**Affiliations:** Department of Traditional Chinese Medicine, Beijing Boai Hospital, China Rehabilitation Research Center, School of Rehabilitation Medicine, Capital Medical University, Beijing, China

**Keywords:** blood selenium, stroke, all-cause mortality, interaction effect, NHANES blood selenium levels and stroke history on all-cause mortality

## Abstract

**Aim:**

The study aimed to investigate the interaction effect between blood selenium levels and stroke history on all-cause mortality.

**Methods:**

In this retrospective cohort study, participant data were obtained from the National Health and Nutrition Examination Survey (NHANES) 2011–2018. The covariates were screened via the backward selection method in weighted univariate and multivariate Cox regression models. Weighted univariate and multivariate Cox regression models were conducted to investigate the association of blood selenium and stroke history with all-cause mortality. The results were expressed as hazard ratios (HRs) and 95% confidence intervals (CIs). The synergy index (SI) was used to assess the assistive interaction. The association was further explored in different gender groups.

**Results:**

Totally, 8,989 participants were included, of whom 861 (9.57%) died. Participants with blood selenium ≥192.96 ug/L were associated with lower odds of all-cause mortality (HR = 0.70, 95% CI: 0.58–0.84), whereas those with a stroke history were associated with a higher risk of all-cause mortality (HR = 1.57, 95% CI: 1.15–2.16). Compared to participants with blood selenium ≥192.96 ug/L and non-stroke history, participants with both blood selenium < 192.96 ug/L and stroke history had a higher all–cause mortality risk (HR = 2.31, 95% CI: 1.62–3.29; SI = 0.713, 95% CI: 0.533–0.952). All participants with blood selenium < 192.96 ug/L and stroke history were related to higher all–cause mortality risk (HR = 1.61, 95% CI: 1.21–2.13). In males, the interaction effect of blood selenium and stroke history on all–cause mortality (HR = 2.27, 95% CI: 1.50–3.46; SI = 0.651, 95% CI: 0.430–0.986) increased twice.

**Conclusion:**

Blood selenium and stroke history have an interaction effect on all-cause mortality. Increasing selenium-rich food or supplement intake, especially for individuals with a stroke history, may improve poor prognosis.

## Introduction

Stroke, a neurological emergency, is the second leading cause of death and a major contributor to disability worldwide (1). Stroke affects 13.7 million people and causes 5.5 million deaths (2). Stroke is also responsible for about 140,000 deaths in the U.S. every year, which is about one out of every 20 deaths in the country (3). Stroke history is an independent risk factor for poor prognosis in ischemic stroke patients (4). Oxidative stress and inflammation play significant roles in the pathogenesis of stroke (5–7).

Selenium, an essential trace element, plays a critical role in various physiologic processes, including oxidative stress, thyroid hormone metabolism, and immune function (8, 9). Lower circulating selenium levels have been linked to an elevated risk of cardiovascular disease, increased risk of ischemic stroke, and all-cause mortality (10). In patients with heart failure, blood selenium was independently associated with a 50% higher mortality rate (11). A lower concentration of selenium could increase the risk of ischemic stroke (12). And Wang et al. reported that plasma selenium was inversely associated with the risk of a first ischemic stroke (13). Zhao et al. (14) also found a negative relationship between blood selenium levels and stroke. In addition, the modifying effect of selenium was observed in metabolic disease, cardiovascular disease (CVD), and neurologic symptoms (15–17). We hypothesize that blood selenium level and stroke history may have an interaction with the long-term prognosis of participants.

Thus, this study aimed to investigate the interaction effect of blood selenium and stroke on all-cause mortality. The findings of this study will contribute to existing knowledge on the role of blood selenium in stroke prognosis and provide guidelines for the development of targeted interventions to improve outcomes for individuals with stroke histories.

## Methods

### Study design and population

The data for this retrospective cohort study were extracted from the National Health and Nutritional Examination Survey (NHANES) between 2011 and 2018. NHANES is a comprehensive survey that provides valuable data on the health and nutritional status of individuals in the U.S. These secondary survey data are usually selected through a complex sampling design to collect information through interviews, physical examinations, and laboratory tests. The protocols of NHANES have been reviewed and approved by the National Center for Health Statistics (NCHS) Ethics Review Board. All participants signed written informed consent. Our study was exempted from screening by the Ethics Committee of Beijing Boai Hospital. Individuals aged ≥ 45 years were included in the database. Participants were excluded if they met any of the following criteria: (1) missing information about stroke, (2) missing data about blood selenium, (3) missing survival data, and (4) missing important co-variables.

### Blood selenium assessment

Whole blood samples were collected in vacuum containers and transported to the National Center for Environmental Health under appropriate frozen conditions (−20°C). After the dilution treatment, blood selenium levels were measured using inductively coupled plasma dynamic reaction cell mass spectrometry. Two groups were divided based on the median blood selenium level.

### Definition of a stroke

Stroke was defined as the question “Has a doctor or other health professional ever told you that you had a stroke?” Participants with a response of “yes” were considered to have a stroke history (18).

### Covariate

The following covariates were included: age, gender, race, poverty income ratio (PIR), smoking, alcohol consumption, physical activity, chronic kidney disease (CKD), anticoagulants, and cardiovascular agents. Information on age, gender, race, PIR, smoking, alcohol consumption, physical activity, and medication use was collected from family interviews and mobile examination centers using standardized questionnaires. PIR, calculated by the family income ratio to the federal poverty threshold, was used to assess the socioeconomic status of participants (19). Smoking was defined as the answer “yes” to the question “Smoked at least 100 cigarettes in life” (20). CKD was defined as an estimated glomerular filtration rate < 60 mL/min/1.73m^2^ or urine albumin-to-creatinine ratio ≥ 30 mg/g (21). Cardiovascular agents include agents of antiadrenergic, antianginal, antiarrhythmic, inotropic, miscellaneous cardiovascular, vasodilators, vasopressors, angiotensin II inhibitors, aldosterone receptor antagonists, renin inhibitors, neprilysin inhibitors, and antihypertensives.

### Outcomes

The study's outcome was all-cause mortality. Mortality status, cause of death, and follow-up time were determined based on the National Death Index (NDI), which can be downloaded from the NCHS website at https://www.cdc.gov/nchs/index.htm. Included participants were followed up until 31 December 2019. The International Classification of Diseases was utilized to ascertain the cause of death.

### Statistical analysis

Appropriate weighting (SDMVPSU^1^ SDMVSTRA^2^ WTMEC2YR^3^) was carried out in the statistical analysis. Continuous variables were presented as mean and standard error (SE), and differences among groups were analyzed by a weighted *t*-test. Categorical variables were presented as numbers and percentages, and differences among groups were analyzed by the chi-square test and Fisher's exact test. The potential covariates were selected through weighted univariate and multivariate Cox regression models. Weighted univariate and multivariate Cox regression models were conducted to investigate the interaction effect of blood selenium and stroke on all-cause mortality with a Hazard Ratio (HR) and 95% Confidence Interval (CI). Model 1 was a crude model. Model 2 was adjusted for age, gender, race, PIR, smoking, alcohol consumption, physical activity, CKD, anticoagulants, and cardiovascular agents. The synergy index (SI) was used to assess the additive interaction. When the CI of Selenium contained 1, there was no additive interaction effect—SI=(HR11-1)/[(HR01-1) (HR10-1)]. HR01 and HR10 indicate that only exposure a occurs or only exposure b occurs, and HR 11 indicates that two exposures occur simultaneously. To further explore the association, subgroup analyses stratified by gender were performed. As no criteria for the division of serum selenium levels were available, we applied the median (192.96 ug/L) to divide the whole blood selenium level. Sensitivity analyses were also performed to detect the availability of the median as a cut-off value, the results are shown in the Supplementary material. All statistical analyses were conducted using R^4^, version 4.2.3 and SAS^5^ version 9.4. Results were considered statistically significant with a two-sided *P* < 0.05.

## Results

### Characteristics of participants

**Figure 1:**
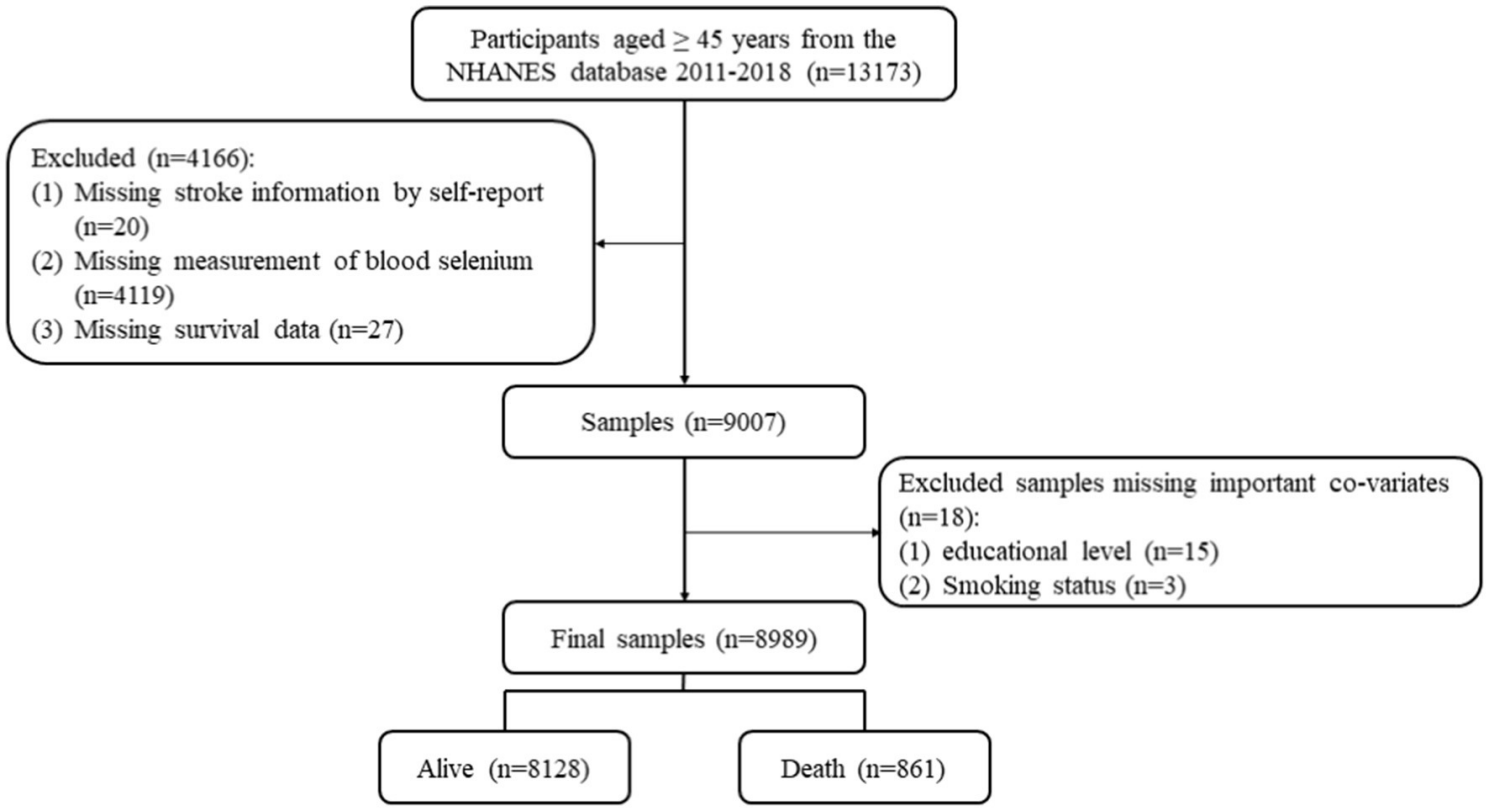
The screening process for participants.

Figure 1 shows the screening process for participants. A total of 13,173 participants aged ≥ 45 years were extracted from the database in 2011–2018. Then, participants were excluded with missing information on stroke history (n = 20), blood selenium level (*n* = 4,119), and survival data (*n* = 27). Individuals who missed important covariates (*n* = 18) were also excluded. Finally, 8,989 participants were included in the final analysis.

The characteristics of the included individuals are shown in Table 1. Totally, 6.52% (*n* = 586) of the participants had a stroke history. During a median follow-up of 52 months, 861 individuals died. Statistical differences were observed in age, gender, race, educational level, PIR, heavy alcohol drinking, energy intake, heart disease, dyslipidemia, CKD, height, weight, body mass index, diastolic blood pressure, albumin, creatinine, uric acid, anticoagulants, antiplatelet agents, stroke, and all-cause mortality between the blood selenium < 192.96 ug/L and the blood selenium ≥192.96 ug/L groups (all *P* < 0.05).

**Table 1:** Characteristics of participants.

**Variables**	**Total (*n* = 8,989)**	**Blood selenium**		***P***
		<**192.96 ug/L (*****n*** = **4,719)**	≥**192.96 ug/L (*****n*** = **4,270)**	
Age, years, *n* (%)				0.001^#^
< 60	3,769 (50.36)	1,876 (47.66)	1,893 (53.07)	
≥60	5,220 (49.64)	2,843 (52.34)	2,377 (46.93)	
Gender, *n* (%)				< 0.001^#^
Female	4,584 (53.09)	2,543 (57.03)	2,041 (49.15)	
Male	4,405 (46.91)	2,176 (42.97)	2,229 (50.85)	
Race, *n* (%)				0.001^#^
Non-Hispanic white	3,524 (71.68)	1,827 (70.45)	1,697 (72.90)	
Non-Hispanic black	2,099 (9.77)	1,246 (11.50)	853 (8.04)	
Mexican American	1,046 (5.68)	530 (5.75)	516 (5.62)	
Others	2,320 (12.87)	1,116 (12.30)	1,204 (13.44)	
Educational level, *n* (%)				< 0.001^#^
Less than high school	2,261 (14.84)	1,306 (17.12)	955 (12.54)	
High school	2,071 (23.58)	1,114 (24.49)	957 (22.68)	
Above high school	4,657 (61.58)	2,299 (58.39)	2,358 (64.78)	
PIR, *n* (%)				< 0.001^#^
< 2	3,798 (28.21)	2,110 (30.97)	1,688 (25.44)	
≥2	4,229 (63.37)	2,089 (60.16)	2,140 (66.58)	
Unknown	962 (8.42)	520 (8.87)	442 (7.98)	
Smoking, *n* (%)				0.948^#^
No	4,765 (53.04)	2,440 (53.08)	2,325 (52.99)	
Yes	4,224 (46.96)	2,279 (46.92)	1,945 (47.01)	
Heavy alcohol drinking, *n* (%)				< 0.001^#^
No	4,270 (54.63)	2,124 (51.56)	2,146 (57.70)	
Yes	744 (10.69)	377 (10.18)	367 (11.20)	
Unknown	3,975 (34.68)	2,218 (38.26)	1,757 (31.10)	
Physical activity, *n* (%)				0.063^#^
Low level	6,713 (70.27)	3,606 (71.69)	3,107 (68.84)	
High level	2276 (29.73)	1113 (28.31)	1163 (31.16)	
Energy intake, kcal/day, Mean ± SE	2,009.29 ± 12.99	1,973.47 ± 16.08	2,045.08 ± 17.42	0.002^*^
Age for diagnosis of stroke, years, Mean ± SE	57.35 ± 0.65	58.07 ± 1.03	56.26 ± 1.47	0.401^*^
Heart disease, *n* (%)				0.019^#^
No	7,979 (90.11)	4,141 (89.03)	3,838 (91.19)	
Yes	1,010 (9.89)	578 (10.97)	432 (8.81)	
Hypertension, *n* (%)				0.717^#^
No	2,506 (33.09)	1,284 (32.78)	1,222 (33.41)	
Yes	6,483 (66.91)	3,435 (67.22)	3,048 (66.59)	
Diabetes, *n* (%)				0.683^#^
No	6,283 (76.81)	3,338 (77.06)	2,945 (76.55)	
Yes	2,706 (23.19)	1,381 (22.94)	1,325 (23.45)	
Dyslipidemia, *n* (%)				< 0.001^#^
No	1,645 (18.18)	981 (20.72)	664 (15.65)	
Yes	7,344 (81.82)	3,738 (79.28)	3,606 (84.35)	
CKD, *n* (%)				0.002^#^
No	6,639 (78.35)	3,399 (76.23)	3,240 (80.46)	
Yes	2,350 (21.65)	1,320 (23.77)	1,030 (19.54)	
Depression, *n* (%)				0.121^#^
No	8,204 (92.41)	4,269 (91.69)	3,935 (93.12)	
Yes	785 (7.59)	450 (8.31)	335 (6.88)	
Obesity, *n* (%)				0.260^#^
No	5,354 (59.53)	2,763 (60.47)	2,591 (58.59)	
Yes	3,635 (40.47)	1,956 (39.53)	1,679 (41.41)	
BMI, kg/m^2^, mean ± SE	29.61 ± 0.15	29.35 ± 0.18	29.87 ± 0.19	0.031^*^
SBP, mmHg, mean ± SE	128.15 ± 0.40	128.55 ± 0.55	127.77 ± 0.42	0.169^*^
DBP, mmHg, mean ± SE	72.10 ± 0.30	71.14 ± 0.39	73.06 ± 0.31	< 0.001^*^
Albumin, g/dL, mean ± SE	4.18 ± 0.01	4.13 ± 0.01	4.24 ± 0.01	< 0.001^*^
Creatinine, mg/dL, mean ± SE	0.92 ± 0.01	0.94 ± 0.01	0.90 ± 0.00	< 0.001^*^
Uric acid, mg/dL, mean ± SE	5.47 ± 0.02	5.42 ± 0.03	5.51 ± 0.03	0.026^*^
Anticoagulants, *n* (%)				0.005^#^
No	8,670 (96.87)	4,505 (96.10)	4,165 (97.64)	
Yes	319 (3.13)	214 (3.90)	105 (2.36)	
Antiplatelet agent, *n* (%)				0.011^#^
No	8,526 (95.64)	4,453 (94.74)	4,073 (96.53)	
Yes	463 (4.36)	266 (5.26)	197 (3.47)	
Cardiovascular agent, *n* (%)				0.161^#^
No	3,825 (48.55)	1,930 (47.50)	1,895 (49.61)	
Yes	5,164 (51.45)	2,789 (52.50)	2,375 (50.39)	
Stroke, *n* (%)				< 0.001^#^
No	8,403 (94.99)	4,358 (93.93)	4,045 (96.05)	
Yes	586 (5.01)	361 (6.07)	225 (3.95)	
All-cause mortality, *n* (%)				< 0.001^#^
No	8,128 (92.27)	4,174 (90.68)	3,954 (93.85)	
Yes	861 (7.73)	545 (9.32)	316 (6.15)	

^*^t- test; ^#^chi-square test; SE, standard error.

PIR, poverty income ratio; CKD, chronic kidney disease; BMI, body mass index; SBP, systolic blood pressure; DBP, diastolic blood pressure.

### Associations of blood selenium and stroke history with all-cause mortality

The associations between blood selenium, stroke, and all-cause mortality are shown in Table 2. In model 2, covariates were adjusted for age, gender, race, PIR, smoking, physical activity, CKD, anticoagulants, and cardiovascular agents. Compared to those who had blood selenium < 192.26 ug/L, those who had blood selenium ≥192.96 ug/L were associated with lower odds of all-cause mortality (HR = 0.70, 95% CI: 0.58–0.84). Participants with a stroke history were associated with a higher risk of all-cause mortality compared to those without a stroke history (HR = 1.57, 95% CI: 1.15–2.16).

**Table 2:** Associations of blood selenium and stroke history with all-cause mortality.

**Variables**	**Model 1**		**Model 2**	
	**HR (95% CI)**	***P***	**HR (95% CI)**	***P***
Blood selenium	0.99 (0.98–0.99)	< 0.001	0.99 (0.99–1.00)	< 0.001
**Blood selenium**				
< 192.96 ug/L	Ref		Ref	
≥192.96 ug/L	0.63 (0.52–0.75)	< 0.001	0.70 (0.58–0.84)	< 0.001
**Stroke**				
No	Ref		Ref	
Yes	3.77 (2.80–5.06)	< 0.001	1.57 (1.15–2.16)	0.005

Ref, reference; HR, hazard ratio; CI, confidence interval.

Model 1: Crude model.

Model 2: Adjusting age, gender, race, poverty income ratio, smoking, physical activity, chronic kidney disease, anticoagulants, and cardiovascular agents.

### The interaction effect between blood selenium and stroke history on all-cause mortality

**Figure 2:**
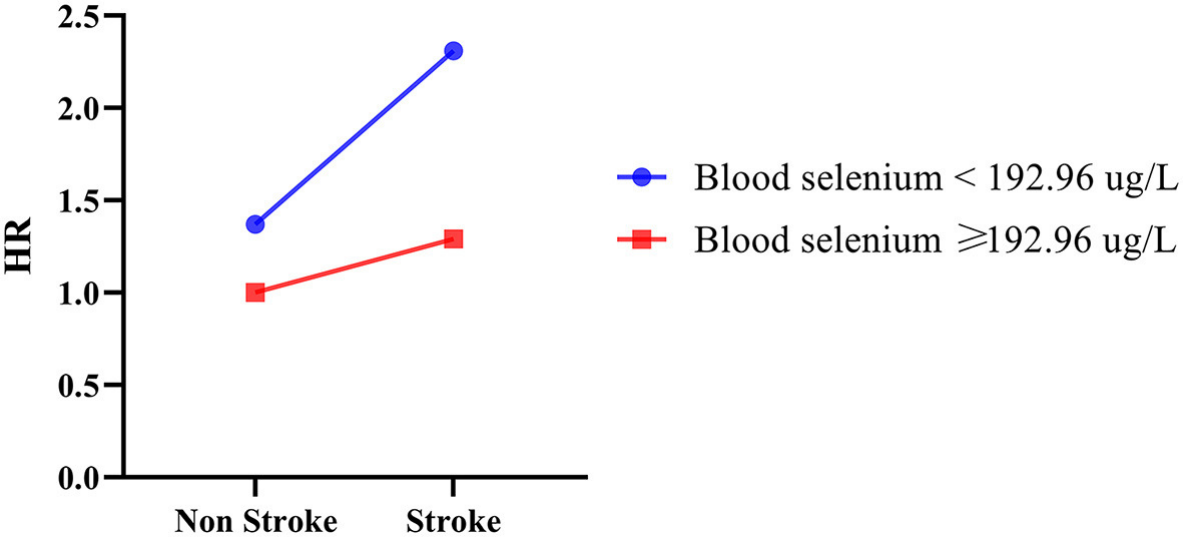
The interaction effect between blood selenium and stroke history on all-cause mortality.

The additive interaction effects of blood selenium and stroke were established, including blood selenium ≥192.96 ug/L and no stroke, blood selenium ≥192.96 ug/L and stroke, blood selenium < 192.26 ug/L and no stroke, and blood selenium < 192.26 ug/L and stroke. Table 3 and Figure 2 show more detail on interaction effect terms. Compared to participants with blood selenium ≥192.96 ug/L and non-stroke history, participants with blood selenium < 192.26 ug/L and stroke history were associated with a higher all-cause mortality risk (HR = 2.31, 95% CI: 1.62–3.29). The SI was 0.713 (95% CI: 0.533–0.952), indicating an interaction effect was observed between blood selenium and stroke history on all-cause mortality. We further investigated the association between stroke history and all-cause mortality at different blood selenium levels. Stroke history remained associated with a higher all-cause mortality risk in participants with blood selenium < 192.96 ug/L (HR = 1.61, 95% CI: 1.21–2.13) (Table 4).

**Table 3:** The interaction effect of blood selenium and stroke history on all-cause mortality.

**Variables**	**Model 1**		**Model 2**	
	**HR (95% CI)**	***P***	**HR (95% CI)**	***P***
**Groups**				
Blood selenium ≥ 192.96 ug/L and Non-stroke	Ref		Ref	
Blood selenium ≥ 192.96 ug/L and Stroke	3.03 (1.91–4.81)	< 0.001	1.29 (0.78–2.13)	0.314
Blood selenium < 192.96 ug/L and Non-stroke	1.50 (1.21–1.85)	< 0.001	1.37 (1.11–1.68)	0.003
Blood selenium < 192.96 ug/L and Stroke	6.02 (4.31–8.40)	< 0.001	2.31 (1.62–3.29)	< 0.001
SI	0.668 (0.564–0.792)		0.713 (0.533–0.952)	

Ref, reference; HR, hazard ratio; CI, confidence interval; SI, the synergy index.

Model 1: Crude model.

Model 2: Adjusting age, gender, race, poverty income ratio, smoking, physical activity, chronic kidney disease, anticoagulants, and cardiovascular agents.

**Table 4:** Associations between stroke history and all-cause mortality at different blood selenium levels.

**Variables**	**Model 1**		**Model 2**	
	**HR (95% CI)**	***P***	**HR (95% CI)**	***P***
**Blood selenium**<**192.96 ug/L**			
**Stroke**			
No	Ref		Ref	
Yes	3.95 (2.96–5.27)	< 0.001	1.61 (1.21–2.13)	0.001
**Blood selenium** ≥**192.96 ug/L**			
**Stroke**			
No	Ref		Ref	
Yes	3.06 (1.91–4.89)	< 0.001	1.44 (0.89–2.31)	0.134

Ref, reference; HR, hazard ratio; CI, confidence interval.

Model 1: Crude model.

Model 2: Adjusting age, gender, race, poverty income ratio, smoking, physical activity, chronic kidney disease, anticoagulants, and cardiovascular agents.

### Interaction effect of blood selenium and stroke history on all-cause mortality in participants with different gender groups

**Figure 3:**
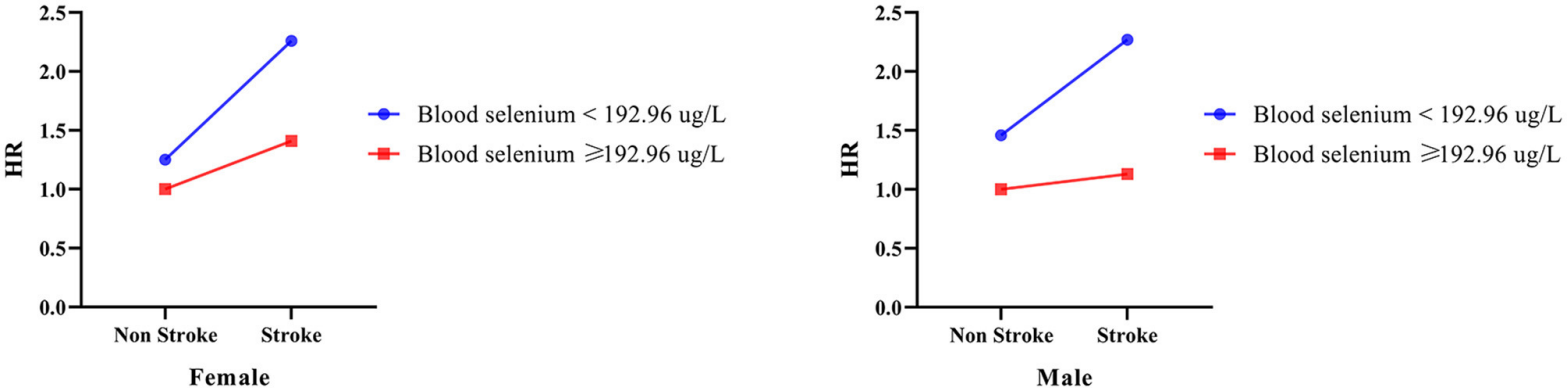
The interaction effect between blood selenium and stroke history on all-cause mortality in different gender populations.

As summarized in Table 5 and Figure 3, further analysis was performed to investigate the interaction effect of blood selenium and stroke history on all-cause mortality in populations of different genders. Participants with a stroke history and blood selenium < 192.26 ug/L were associated with an increased risk of all-cause mortality (HR = 2.27, 95% CI: 1.50–3.46). In addition, the interaction effect of blood selenium and stroke history on all-cause mortality existed in males (SI = 0.651, 95% CI: 0.430–0.986).

**Table 5:** The interaction effect of blood selenium and stroke history on all-cause mortality in different gender groups.

**Variables**	**Female**		**Male**	
	**HR (95% CI)**	***P***	**HR (95% CI)**	***P***
**Groups**				
Blood selenium ≥ 192.96 ug/L and Non-stroke	Ref		Ref	
Blood selenium ≥ 192.96 ug/L and Stroke	1.41 (0.70–2.82)	0.336	1.13 (0.64–1.99)	0.666
Blood selenium < 192.96 ug/L and Non-stroke	1.25 (0.91–1.73)	0.165	1.46 (1.14–1.86)	0.003
Blood selenium < 192.96 ug/L and Stroke	2.26 (1.38–3.70)	0.001	2.27 (1.50–3.46)	< 0.001
SI	0.678 (0.408–1.125)		0.651 (0.430–0.986)	

Ref, reference; HR, hazard ratio; CI, confidence interval; SI, the synergy index.

Adjusting age, race, poverty income ratio, smoking, physical activity, chronic kidney disease, anticoagulants, and cardiovascular agents.

## Discussion

The study aimed to investigate the interaction effect of blood selenium and stroke history on all-cause mortality. Individuals with both low blood selenium and stroke histories have a higher all-cause mortality risk compared to those with neither condition. An interaction effect was found between blood selenium and stroke history on all-cause mortality. Furthermore, the interaction effect of blood selenium and stroke history on all-cause mortality was also observed in males.

Selenium is an essential nutrient for normal human physiological processes. Xing et al. (22) reported that blood selenium was associated with a lower risk of CVD mortality in heart failure. A meta-analysis also found that high blood selenium levels in the body were associated with a decreased risk of CVD incidence and mortality (23). Similarly, Zhao et al. (24) found higher selenium concentrations were related to lower all-cause mortality. Stroke is an important cause of death in the U.S (25). Stroke history was an independent risk factor for poor prognosis in ischemic stroke patients (4). A Japanese study found that older adults who have experienced a stroke could have a lower life expectancy (26). Our study found higher odds of all-cause mortality in individuals with low blood selenium and stroke histories which stresses the potential synergistic influence of these two conditions on health outcomes. Our findings were in concordance with previous studies that have reported an individual association between low selenium levels and increased mortality risk, as well as an association between stroke history and a higher incidence of mortality (10, 27, 28). However, the novelty of our study lies in showing the interactive effect, emphasizing the importance of assessing these conditions concurrently.

In addition, the findings in subgroups suggested that the interaction effect of blood selenium and stroke history also existed in males. Li et al. (29) found a relationship between serum selenium and all-cause mortality in both genders, and higher mortality in women with ischemic stroke (30). The difference may be related to the limited sample size and lower blood selenium levels in males.

To comprehend the underlying mechanisms contributing to the higher odds of all-cause mortality in individuals with low blood selenium and stroke histories, it is imperative to explore the biological pathways implicated. Selenium, as an essential immune nutrient, also plays a pivotal role in anti-oxidative mechanisms and thyroid hormone metabolism (31). In participants with stroke histories, the oxidative stress and inflammatory cascades that ensure this may be exacerbated in individuals with suboptimal selenium levels (32). The compromised antioxidant capacity, coupled with an impaired ability to modulate inflammation, may synergistically contribute to an augmented susceptibility to adverse outcomes in the presence of a stroke history. Moreover, the observed interaction effect may also be rooted in selenium's influence on cardiovascular health. Selenium has been implicated in endothelial function, blood clotting, and vascular integrity, all of which are critical in the aftermath of a stroke event (33). In individuals with low blood selenium levels, compromised vascular resilience may synergize with the deleterious effects of stroke, leading to an enhanced risk of mortality (34).

And selenium-regulated cardiomyocyte apoptosis (35). Unraveling these intricate pathways is essential for a comprehensive understanding of the observed interaction effects.

Healthcare providers should monitor blood selenium levels in stroke patients, considering it a potential modifiable factor to improve long-term outcomes. Incorporating selenium-rich foods or supplements in at-risk populations, especially those with stroke, may serve as a mitigating factor against poor prognosis. Brazil nuts are one of the most abundant sources of selenium, with one nut providing nearly 100% selenium of the recommended daily intake (36). Other nuts, such as walnuts and almonds also contain selenium, albeit in smaller amounts (37). Seafood, particularly tuna, shrimp, and sardines, is another excellent source of selenium (38). For those who prefer plant-based options, whole grains like wheat and rice, as well as legumes such as lentils and chickpeas, can contribute to selenium intake (39). Incorporating these foods into daily meals can help individuals achieve desired blood selenium levels. It is essential to note that while selenium is crucial for health, excessive intake can lead to toxicity. Therefore, it is advisable to consult with a healthcare provider or nutritionist before significantly altering selenium intake, especially through supplements.

We were unable to differentiate hemorrhagic and ischemic stroke and their duration from the secondary data used, which limited our ability to investigate and compare stroke subtypes and blood selenium with all-cause mortality. Given the retrospective nature of this study, some important information that could have been beneficial to this study might not have been provided. Finally, since information on stroke was self-reported by participants, this may lead to some gaps in the recall of information.

## Conclusion

Our study suggested an interaction effect between blood selenium and stroke on all-cause mortality. The complex interplay of these factors necessitates further research to delineate the underlying mechanisms and inform targeted interventions, ultimately contributing to more effective strategies for reducing mortality risk in individuals with low blood selenium levels and stroke.

## Data availability statement

Publicly available datasets were analyzed in this study. This data can be found here: NHANES database (https://wwwn.cdc.gov/nchs/nhanes/).

## Ethics statement

Ethical review and approval was not required for the study on human participants in accordance with the local legislation and institutional requirements. Written informed consent from the patients/participants or patients/participants' legal guardian/next of kin was not required to participate in this study in accordance with the national legislation and the institutional requirements.

## Author contributions

YL: Conceptualization, Project administration, Supervision, Writing – original draft, Writing – review & editing. LL: Data curation, Formal analysis, Investigation, Methodology, Writing – review & editing. ZY: Data curation, Formal analysis, Investigation, Methodology, Writing – review & editing. JX: Conceptualization, Project administration, Supervision, Writing – review & editing.

## Footnotes

^1^The masked variance unit pseudo-stratum was SDMVSTRA, and the masked variance unit pseudoprimary sampling units was SDMVPSU.

^2^The confidence interval (CI) was applied for evaluating the reliability of an estimate.

^3^The 2-year sample weights (WTINT2YR, WTMEC2YR) should be used for all NHANES 2011.2012, 2013-2014, 2015-2016 and 2017-2018 analyses.

^4^ https://www.r-project.org/

^5^ https://www.rproject.org/about.html

## References

[1] Kuriakose D Xiao Z. Pathophysiology and treatment of stroke: present status and future perspectives. Int J Mol Sci. (2020) 21:7609. 10.3390/ijms21207609 33076218 PMC7589849

[2] Saini V Guada L Yavagal DR. Global epidemiology of stroke and access to acute ischemic stroke interventions. Neurology. (2021) 97:S6–S16. 10.1212/WNL.0000000000012781 34785599

[3] Barthels D Das H. Current advances in ischemic stroke research and therapies. Biochim Biophys Acta Mol Basis Dis. (2020) 1866:165260. 10.1016/j.bbadis.2018.09.012 31699365 PMC6981280

[4] Qin H Wang P Zhang R Yu M Zhang G Liu G. Stroke history is an independent risk factor for poor prognosis in ischemic stroke patients: results from a large nationwide stroke registry. Curr Neurovasc Res. (2020) 17:487–94. 10.2174/1567202617666200817141837 32807054 PMC8493791

[5] Orellana-Urzúa S Rojas I Líbano L Rodrigo R. Pathophysiology of ischemic stroke: role of oxidative stress. Curr Pharm Des. (2020) 26:4246–60. 10.2174/1381612826666200708133912 32640953

[6] Simats A Liesz A. Systemic inflammation after stroke: implications for post-stroke comorbidities. EMBO Mol Med. (2022) 14:e16269. 10.15252/emmm.202216269 35971650 PMC9449596

[7] Candelario-Jalil E Dijkhuizen RM Magnus T. Neuroinflammation, stroke, blood-brain barrier dysfunction, and imaging modalities. Stroke. (2022) 53:1473–86. 10.1161/STROKEAHA.122.036946 35387495 PMC9038693

[8] Hariharan S Dharmaraj S. Selenium and selenoproteins: it's role in regulation of inflammation. Inflammopharmacology. (2020) 28:667–95. 10.1007/s10787-020-00690-x 32144521 PMC7222958

[9] Cai Z Zhang J Li H. Selenium, aging and aging-related diseases. Aging Clin Exp Res. (2019) 31:1035–47. 10.1007/s40520-018-1086-7 30511318

[10] Xiang S Dai Z Man C Fan Y. Circulating selenium and cardiovascular or all-cause mortality in the general population: a meta-analysis. Biol Trace Elem Res. (2020) 195:55–62. 10.1007/s12011-019-01847-8 31368032

[11] Bomer N Grote Beverborg N Hoes MF Streng KW Vermeer M Dokter MM. Selenium and outcome in heart failure. Eur J Heart Fail. (2020) 22:1415–23. 10.1002/ejhf.1644 31808274 PMC7540257

[12] Wen Y Huang S Zhang Y Zhang H Zhou L Li D. Associations of multiple plasma metals with the risk of ischemic stroke: a case-control study. Environ Int. (2019) 125:125–34. 10.1016/j.envint.2018.12.037 30716572

[13] Wang Z Hu S Song Y Liu L Huang Z Zhou Z. Association between plasma selenium and risk of ischemic stroke: a community-based, nested, and case-control study. Front Nutr. (2022) 9:1001922. 10.3389/fnut.2022.1001922 36466415 PMC9716699

[14] Zhao K Zhang Y Sui W. Association between blood selenium levels and stroke: a study based on the NHANES (2011-2018). Biol Trace Elem Res. (2023) 202:25–33. 10.1007/s12011-023-03649-5 37004705

[15] Werder EJ Engel LS Curry MD Sandler DP. Selenium modifies associations between multiple metals and neurologic symptoms in Gulf states residents. Environ Epidemiol Phila Pa. (2020) 4:e115. 10.1097/EE9.0000000000000115 33336134 PMC7727467

[16] Park K Seo E. Association between toenail mercury and metabolic syndrome is modified by selenium. Nutrients. (2016) 8:424. 10.3390/nu8070424 27420091 PMC4963900

[17] Zhang C Deng Y Lei Y Zhao J Wei W Li Y. Effects of selenium on myocardial apoptosis by modifying the activity of mitochondrial STAT3 and regulating potassium channel expression. Exp Ther Med. (2017) 14:2201–5. 10.3892/etm.2017.4716 28962142 PMC5609099

[18] Ye J Hu Y Chen X Yin Z Yuan X Huang L. Association between the weight-adjusted waist index and stroke: a cross-sectional study. BMC Public Health. (2023) 23:1689. 10.1186/s12889-023-16621-8 37658310 PMC10472709

[19] Murphy R Marshall K Zagorin S Devarshi PP Hazels Mitmesser S. Socioeconomic inequalities impact the ability of pregnant women and women of childbearing age to consume nutrients needed for neurodevelopment: an analysis of NHANES 2007–2018. Nutrients. (2022) 14:3823. 10.3390/nu14183823 36145198 PMC9500720

[20] Zhu D Zhao G Wang X. Association of smoking and smoking cessation with overall and cause-specific mortality. Am J Prev Med. (2021) 60:504–12. 10.1016/j.amepre.2020.11.003 33745522

[21] Murphy D McCulloch CE Lin F Banerjee T Bragg-Gresham JL Eberhardt MS. Trends in prevalence of chronic kidney disease in the United States. Ann Intern Med. (2016) 165:473–81. 10.7326/M16-0273 27479614 PMC5552458

[22] Xing X Xu M Yang L Shao C Wang Y Qi M. Association of selenium and cadmium with heart failure and mortality based on the National Health and Nutrition Examination Survey. J Hum Nutr Diet. (2023) 36:1496–506. 10.1111/jhn.13107 36321401

[23] Kuria A Tian H Li M Wang Y Aaseth JO Zang J. Selenium status in the body and cardiovascular disease: a systematic review and meta-analysis. Crit Rev Food Sci Nutr. (2021) 61:3616–25. 10.1080/10408398.2020.1803200 32799545

[24] Zhao S Wang S Yang X Shen L. Dose–response relationship between multiple trace elements and risk of all-cause mortality: a prospective cohort study. Front Nutr. (2023) 10:1205537. 10.3389/fnut.2023.1205537 37533572 PMC10391637

[25] Wu H Le Couteur DG Hilmer SN. Mortality trends of stroke and dementia: changing landscapes and new challenges. J Am Geriatr Soc. (2021) 69:2888–98. 10.1111/jgs.17322 34133024

[26] Chiu C-T Yong V Chen H-W Saito Y. Disabled life expectancy with and without stroke: a 10-year Japanese prospective cohort study. Qual Life Res Int J Qual Life Asp Treat Care Rehabil. (2019) 28:3055–64. 10.1007/s11136-019-02246-1 31309398

[27] Singh R-J Chen S Ganesh A Hill MD. Long-term neurological, vascular, and mortality outcomes after stroke. Int J Stroke Off J Int Stroke Soc. (2018) 13:787–96. 10.1177/1747493018798526 30160619

[28] Mathisen SM Dalen I Larsen JP Kurz M. Long-term mortality and its risk factors in stroke survivors. J Stroke Cerebrovasc Dis Off J Natl Stroke Assoc. (2016) 25:635–41. 10.1016/j.jstrokecerebrovasdis.2015.11.039 26738815

[29] Li J Lo K Shen G Feng Y-Q Huang Y-Q. Gender difference in the association of serum selenium with all-cause and cardiovascular mortality. Postgrad Med. (2020) 132:148–55. 10.1080/00325481.2019.1701864 31810414

[30] Roy-O'Reilly M McCullough LD. Age and sex are critical factors in ischemic stroke pathology. Endocrinology. (2018) 159:3120–3131. 10.1210/en.2018-00465 30010821 PMC6963709

[31] Silvestrini A Mordente A Martino G Bruno C Vergani E Meucci E. The role of selenium in oxidative stress and in Nonthyroidal Illness Syndrome (NTIS): an overview. Curr Med Chem. (2020) 27:423–49. 10.2174/0929867325666180201111159 29421998

[32] Zou L Han R. Inflammatory response and immune regulation in brain-heart interaction after stroke. Cardiovasc Ther. (2022) 2022:2406122. 10.1155/2022/2406122 36474712 PMC9683992

[33] Shalihat A Hasanah AN Mutakin N Lesmana R Budiman A Gozali D. The role of selenium in cell survival and its correlation with protective effects against cardiovascular disease: a literature review. Biomed Pharmacother Biomedecine Pharmacother. (2021) 134:111125. 10.1016/j.biopha.2020.111125 33341057

[34] Gać P Czerwińska K Macek P Jaremków A Mazur G Pawlas K. The importance of selenium and zinc deficiency in cardiovascular disorders. Environ Toxicol Pharmacol. (2021) 82:103553. 10.1016/j.etap.2020.103553 33238203

[35] Shimada BK Alfulaij N Seale LA. The impact of selenium deficiency on cardiovascular function. Int J Mol Sci. (2021) 22:10713. 10.3390/ijms221910713 34639053 PMC8509311

[36] Cardoso BR Duarte GBS Reis BZ Cozzolino SMF: Brazil nuts: nutritional composition health benefits and safety aspects. Food Res Int. (2017) 100:9–18. 10.1016/j.foodres.2017.08.036 28888463

[37] Moskwa J Naliwajko SK Puścion-Jakubik A Soroczyńska J Socha K Koch W Markiewicz-Żukowska R. In vitro assessment of the bioaccessibility of Zn, Ca, Mg, and Se from various types of nuts. Foods. (2023) 12:4453. 10.3390/foods12244453 38137257 PMC10742998

[38] Olmedo P Hernández AF Pla A Femia P Navas-Acien A Gil F. Determination of essential elements (copper, manganese, selenium and zinc) in fish and shellfish samples. Risk and nutritional assessment and mercury-selenium balance. Food Chem Toxicol. (2013) 62:299–307. 10.1016/j.fct.2013.08.076 24007738

[39] Ciudad-Mulero M Matallana-González MC Cámara M Fernández-Ruiz V Morales P. Antioxidant phytochemicals in pulses and their relation to human health: a review. Curr Pharm Des. (2020) 26:1880–97. 10.2174/1381612826666200203130150 32013818

