# Supplementary material for: Interaction effect between blood selenium levels and stroke history on all-cause mortality: a retrospective cohort study of NHANES
Source: Front Neurol. 2024 Jul 5;15:1404570. doi: 10.3389/fneur.2024.1404570 (PMC11257915; doi:10.3389/fneur.2024.1404570)
Supplement: Supplementary file 1 [file Data_Sheet_1.pdf]

In our study, the whole blood selenium as the indicators for evaluating selenium exposure in the human body. After reviewing the literatures, we only found criteria for the division of serum selenium levels. However, there is a difference in levels between serum selenium and whole blood selenium, and it is not appropriate to use the criteria of serum selenium. In our study, we applied the median (192.96 ug/L) to divide the whole blood selenium level. The median of blood selenium (192.96 ug/L) in our study were similar to another study on blood selenium (191.62 ug/L) [1].

In addition, sensitivity analysis was performed with the cut-off value determined based on the Kaplan-Meier (KM) survival curves. Using the "surv_cutpoint" function in R language and the maximally selected rank statistics method, find the point that makes the most significant difference in the survival curve, and the cutoff value is 180.61 ug/L (Figure 1). And the interaction effect between blood selenium levels and stroke history on all-cause mortality remains observed with the cut-off value of 180.61 ug/L (Table 1).

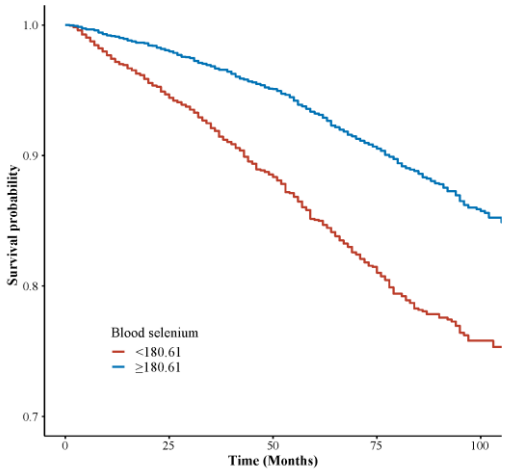

Figure 1 The KM survival curves of blood selenium

Table 1 The interaction effect of blood selenium and stroke history on all-cause mortality.

Variables	Model 1		Model 2	
	HR (95%CI)	*P*	HR (95%CI)	*P*
Blood selenium				
<180.61 ug/L	Ref		Ref	
≥180.61 ug/L	0.47 (0.39-0.56)	<0.001	0.55 (0.46-0.66)	<0.001
Groups				
Blood selenium≥180.61 ug/L & Non Stroke	Ref		Ref	
Blood selenium≥180.61 ug/L & Stroke	3.47 (2.32-5.20)	<0.001	1.53 (1.00-2.34)	0.051
Blood selenium<180.61 ug/L & Non Stroke	2.05(1.68-2.50)	<0.001	1.81(1.49-2.19)	<0.001
Blood selenium<180.61 ug/L & Stroke	6.99 (4.83-10.11)	<0.001	2.59 (1.75-3.83)	<0.001
S	0.709 (0.595 - 0.844)		0.753 (0.583 - 0.974)	

Ref: Reference, HR: Hazard Ratio, CI: Confidence Interval

Model 1 was crude model.

Model 2adjusting age, gender, race, PIR, smoking, physical activity, CKD, anticoagulants, and cardiovascular agent.

Furthermore, we also performed a sensitivity analysis which the cut-off value determined

according to the restricted cubic spline. As blood selenium levels increased, the risk of all-cause

mortality gradually decreased and then leveled off. The corresponding blood selenium level

192.10 ug/L when HR=1 is selected as the cutoff value for classification (Figure 2). The cut-

off value 192.10 ug/L was similar to the median of our 192.96 ug/L. And when cut-off value

was 192.10 ug/L, the interaction effect between blood selenium levels and stroke history on all-

cause mortality remains ol

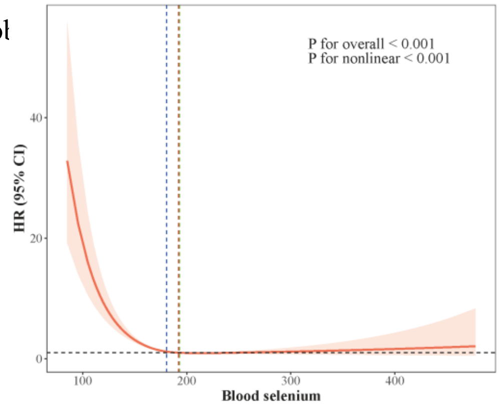

Figure 2 The restricted cubic spline curve of blood selenium

The black dotted line is HR=1; the red vertical dotted line is the blood selenium corresponding to HR=1; blue vertical dashed lines as "surv cutpoint" function to find the cut-off.

Table 2 The interaction effect of blood selenium and stroke history on all-cause mortality.

	Model 1		Model 2	
Variables	HR (95%CI)	*P*	HR (95%CI)	*P*
Blood selenium				
<180.61 ug/L	Ref		Ref	
≥180.61 ug/L	0.47 (0.39-0.56)	<0.001	0.55 (0.46-0.66)	<0.001
Groups				
Blood selenium≥180.61 ug/L & Non Stroke	Ref		Ref	
Blood selenium≥180.61 ug/L & Stroke	3.47 (2.32-5.20)	<0.001	1.53 (1.00-2.34)	0.051
Blood selenium<180.61 ug/L & Non Stroke	2.05(1.68-2.50)	<0.001	1.81(1.49-2.19)	<0.001
Blood selenium<180.61 ug/L & Stroke	6.99 (4.83-10.11)	<0.001	2.59 (1.75-3.83)	<0.001
S	0.709 (0.595 - 0.844)		0.753 (0.583 - 0.974)	

Ref: Reference, HR: Hazard Ratio, CI: Confidence Interval

Model 1 was crude model. Model 2adjusting age, gender, race, PIR, smoking, physical activity, CKD, anticoagulants, and cardiovascular agent.

Ref

[1] Zhang C, Zeng Q, Liu X, et al. Association of Blood Selenium Levels with Diabetes and Heart Failure in American General Adults: a Cross-sectional Study of NHANES 2011-2020 pre. Biol Trace Elem Res. Published online November 23, 2023. doi:10.1007/s12011-023-03933-4IF: 3.9 Q2
